# Coerced syphilis self-testing among men who have sex with men: a nationwide cross-sectional study in China

**DOI:** 10.1186/s12879-022-07476-2

**Published:** 2022-05-23

**Authors:** Peizhen Zhao, Yijia Shi, Cheng Wang

**Affiliations:** 1 STD Control Department, Dermatology Hospital, Southern Medical University, Guangzhou, 510095 China; 2 STD Control Department, Southern Medical University Institute for Global Health, Guangzhou, China; 3 Department of Health, Behavior and Society, Johns Hopkins Bloomberg School of Public Health, Baltimore, USA

**Keywords:** Syphilis, Self-testing, Coercion, Men who have sex with men, National study

## Abstract

**Background:**

Syphilis testing uptake remains low among men who have sex with men (MSM) in many low-and middle-income countries. Syphilis self-testing is an efficacious approach on increasing syphilis testing coverage. However, one unintended consequence is the syphilis self-testing coercion from others (including partners, healthcare providers, friends, etc.). This study aimed to examine the prevalence, pattern and correlates of coerced syphilis self-testing among MSM in China.

**Methods:**

A nationwide online cross-sectional study was conducted from 14 to 28 July 2018. Sociodemographic characteristics, sexual behaviors and coerced syphilis self-testing variables were collected through a questionnaire that targeted MSM in China. Multivariable logistic regression was used to explore associated factors with syphilis self-testing coercion.

**Results:**

Overall, 174 MSM were recruited in this study, 31 men (17.8%) reported ever experiencing syphilis self-testing coercion. The most common types of coercion before syphilis self-testing were verbal abuse (38.7%, 12/31) and threatening to end a relationship (38.7%, 12/31). After obtaining the self-test results, men were mostly subjected to end a relationship (45.2%, 14/31) and threaten of violence (35.5%, 11/31). Multivariable logistic regression indicated that men who used condoms inconsistently in the past three months, ever used substances before or during sex, ever had group sex with males and commercial sex were more likely to experience syphilis self-testing coercion.

**Conclusions:**

Coerced syphilis self-testing was prevalent among MSM in China. Innovative intervention to reduce coerced syphilis self-testing is necessary among MSM in China.

**Supplementary Information:**

The online version contains supplementary material available at 10.1186/s12879-022-07476-2.

## Background

Syphilis continues to be an urgent public health issue globally among men who have sex with men (MSM) [1]. Frequent testing plays a significant role in syphilis prevention and control [2]. According to syphilis guideline in China, sexually active MSM should take syphilis test at least once annually and every 3–6 month for MSM who engaged in risky sexual behaviors [3]. However, syphilis testing uptake remains low among MSM in many low- and middle-income countries (LMIC) [2, 4]. Syphilis self-testing provides a private and more convenient alternative to in clinic testing and results are provided quicker than blood test in clinics [5, 6]. Syphilis self-testing is a process whereby a person can get kits from online testing programs and community-based organization programs, then collect a specimen from finger pricking, perform the test, and interpret the syphilis result themselves [7, 8]. However, one unintended consequence is the syphilis self-testing coercion from others (including partners, healthcare providers, friends, etc.).

Coercion is defined as an individual acting without a voluntary consent, as a result of physical and mental compulsion, threats of violence, and excessive control [9, 10]. Previous studies showed that coercive testing for human immunodeficiency virus (HIV) and syphilis was prevalent among MSM and general population in many LMICs in 2011–2016 [8, 11–14]. Studies conducted among MSM in China, Malawi, and the United States indicated that coerced HIV self-testing (HIVST) can lead to a variety of negative consequences, including unwillingness to take the test again, strained or broken relationships, and the feeling that their human rights have been violated [11, 12, 14, 15]. In recent years, syphilis self-testing has been widely promoted among MSM in China, and can be easily accessed online [8]. A previous study conducted in China found that 48% of MSM who had tested for syphilis used self-testing [8]. However, there is no study on syphilis self-testing coercion. This study aimed to examine the prevalence, pattern and correlates of coerced syphilis self-testing among MSM in China.

## Methods

### Study design and participants

This was a secondary analysis of a nationwide online cross-sectional study conducted from 14 to 28 July 2018 [8]. Link to the online survey was distributed through local health departments and gay-friendly community-based organizations through Weibo (a microblogging platform) and WeChat (a messaging app). Men participated in the survey by clicking on a link, which directed them to a survey website hosted by WenJuanXing (Changsha Haoxing Information Technology, China), a professional online questionnaire platform that can provide anonymous surveys in China.

All potential participants who clicked on the survey link were screened for eligibility after signing an electronic informed consent. Men in China were eligible to participate if they were born biologically as a male, aged 16 or over, and ever engaged in anal or oral sex with a man. In this study, we further restricted to men who had ever self-tested for syphilis during their lifetime. Men self-reported with ever syphilis self-testing experience were requested to upload any one of the following documentations to confirm their syphilis self-testing experience: a copy of the purchase receipt for the syphilis self-testing kit, a screenshot of the transaction record showing the purchase history of a syphilis self-testing kit, or a photograph of their used syphilis self-testing kit. All the participants in this study only need to provide evidence for one test.

To minimize the risk of duplicate participation from the same person, we allowed each survey link to be accessed only by one single device and phone number. All eligible men would receive an incentive of $5 for their time after completing the survey automatically through the WenJuanXing platform.

### Measurements

#### Sociodemographic and behavioral variables

Sociodemographic variables included age, legal heterosexual marriage (never married/ever married/engaged), highest educational attainment (high school or below/junior college/bachelor’s degree or higher), and annual income (< 5000, 5001 to 9000, > 9000 USD). Sexual health and behavior variables included sexual orientation (gay/bisexual/other) and disclosure of sexuality orientation to healthcare providers or family or friends (yes/no), number of male sexual partners, female sexual partners, consistency of condom use in the past 3 months, condom use during last anal sex (yes/no), using substances before or during sex (yes/no), group sex with males (yes/no), commercial sex with men (yes/no), HIV and HIVST history. Substance use was defined as ever using any of the following substances before or during sex: rush, capsule '0', G-point liquid, viagra, heroin/morphine/opium, marijuana methamphetamine/crystal meth, and others. Consistent condom use was defined as always using condoms when engaged in sex, otherwise it was defined as inconsistent condom use. Commercial sex was defined as men who buy or sold sex with men. Syphilis self-testing results were also obtained through questionnaire.

#### Coerced syphilis self-testing variables

Coerced syphilis self-testing was defined as when someone pressures you against your will to do a syphilis self-test by using verbal, physical or psychological threat. Coerced syphilis self-testing variables included times of experiencing syphilis self-testing coercion, the location where syphilis self-testing coercion happened (hotel, your own home, other people's homes, medical and health department, community organization, etc.), relationship with the person who coerced you (fixed male sexual partner, casual male sexual partner, fixed female fixed sexual partner, friends/companions, etc.), types of coercion before syphilis self-testing and after getting syphilis self-testing results (verbal abuse, threatening to end a relationship, threats of violence, psychological pressure, physical violence, etc.) (Additional file 1).

### Statistical analysis

Descriptive analysis was conducted to describe the demographic characteristics, sexual behaviors, types of coercion, and syphilis self-testing experience of each participant. Chi-square tests were used to assess differences in distributions. Univariable and multivariable logistic regression models were constructed to explore factors associated with syphilis self-testing coercion experience. The multivariable model was adjusted for age, legal marital status, highest educational attainment, annual income and sexual orientation. All analyses were conducted using SAS software (V9.4, SAS Institute Inc., Cary, NC).

## Results

Overall, 773 men consented to the survey. Of whom, 59 (8.1%) did not meet the eligibility requirements (13 were female, 2 were younger than 16 years old, 44 men did not engage in anal or oral sex with a man during their lifetime) and 15 duplicated surveys were also excluded. A total of 699 eligible participants from 103 cities of 29 provinces completed this survey, of which, 174 men ever undergone syphilis self-testing during their lifetime, and were finally included in this study. Among those self-testers, 31 men (17.8%) reported ever experiencing syphilis self-testing coercion and 12.6% (22/174) reported a positive result in their most recent syphilis self-test.

### Socio-demographic and sexual behaviors

The median age was 27.0 (18.0–54.0) years old. The majority of participants were between 16 and 35 years old (90.2%), never married (81.0%), self-identified as gay (75.9%), had an annual income less than $9000 USD (59.8%), and had a bachelor's degree and above (52.3%).

Around half of men reported ever using condoms inconsistently with men in the last 3 months (51.6%) and ever using substances before or during sex (55.2%). The majority of participants reported ever disclosed of sexual orientation to family, health providers or friends (77.0%). Around one-fifth of men reported ever having group sex with males (23.6%) and commercial sex (27.0%). Most men reported having ever used HIV self-testing (96.5%). (Table 1).

**Table 1:** Social demographic and sexual behavioral characteristics of participants among men who had syphilis self-testing in China (*N* = 174)

Variable	Total (*N* = 174)	Reported with coercion (*N* = 31)	Reported without coercion (*N* = 143)	*P*
Total		31 (17.8)	143 (82.2)	
*Age*				0.116
16–25	72 (41.4)	10 (32.3)	62 (43.4)	
26–35	85 (48.8)	20 (64.5)	65 (45.5)	
> = 36	17 (9.8)	1 (3.2)	16 (11.1)	
*Legal marital status*				< 0.001
Never married	141 (81.0)	17 (54.8)	124 (86.7)	
Ever married/engaged	33 (19.0)	14 (45.2)	19 (13.3)	
*Highest educational attainment*				0.118
High school or below	38 (21.8)	6 (19.4)	32 (22.3)	
Junior college	45 (25.9)	4 (12.9)	41 (28.7)	
Bachelor's degree or higher	91 (52.3)	21 (67.7)	70 (49.0)	
*Annual income (US$)*				0.360
< $5000	47 (27.0)	7 (22.6)	40 (28.0)	
$5001–9000	57 (32.8)	8 (25.8)	49 (34.2)	
> $9000	70 (40.2)	16 (51.6)	54 (37.8)	
*Sexual orientation*				0.675
Gay	132 (75.9)	22 (71.0)	110 (76.9)	
Bisexual	35 (20.1)	7 (22.6)	28 (19.6)	
Other	7 (4.0)	2 (6.4)	5 (3.5)	
*Disclosure of sexual orientation to family or friends or health provider*				0.317
Yes	134 (77.0)	26 (83.9)	108 (75.5)	
No	40 (23.0)	5 (16.1)	35 (24.5)	
*Number of male sex partners in the past 3 months*				0.025
0	21 (12.0)	0 (0.0)	21 (14.7)	
1	67 (38.5)	9 (29.0)	58 (40.5)	
2–5	80 (46.0)	21 (67.8)	59 (41.3)	
≥ 6	6 (3.5)	1 (3.2)	5 (3.5)	
*Mean*	1.89 ± 1.98	2.29 ± 1.22	1.81 ± 2.10	< 0.001
*Inconsistent condom uses with men in the past 3 months*^a^				0.002
Yes	79 (51.6)	27 (87.1)	52 (42.6)	
No	74 (48.4)	4 (12.9)	70 (57.4)	
*Condom use during last anal sex*				0.002
Yes	55 (31.6)	17 (54.8)	38 (26.6)	
No	119 (68.4)	14 (45.2)	105 (73.4)	
*Ever used substances before or during sex*				< 0.001
Yes	96 (55.2)	27 (87.1)	69 (48.2)	
No	78 (44.8)	4 (12.9)	74 (51.8)	
*Ever had group sex with males*				< 0.001
Yes	41 (23.6)	18 (58.1)	23 (16.1)	
No	133 (76.4)	13 (41.9)	120 (83.9)	
*Ever had commercial sex*				< 0.001
Yes	47 (27.0)	21 (67.7)	26 (18.2)	
No	127 (73.0)	10 (32.3)	117 (81.8)	
*Ever had HIV testing*				0.704
Yes	170 (97.7)	30 (96.8)	140 (97.9)	
No	4 (2.3)	1 (3.2)	3 (2.1)	
*Ever had HIV self-testing*				0.940
Yes	168 (96.5)	30 (96.8)	138 (96.5)	
No	6 (3.5)	1 (3.2)	5 (3.5)	
*Ever had facility syphilis testing*				0.355
Yes	39 (22.4)	5 (16.1)	34 (23.8)	
No	135 (77.6)	26 (83.9)	109 (76.2)	
*Syphilis infection*				< 0.001
Positive	22 (12.6)	11 (35.5)	11 (7.7)	
Negative	152 (87.4)	20 (64.5)	132 (92.3)	

^a^This analysis was restricted to participants who had male sex partners in the past 3 months

Compared to men who had not been subjected to syphilis self-test coercion, men with syphilis self-test coercion experience were more likely to be married (*P* < 0.001), have multiple male sexual partners in the past three months(*P* = 0.025), use condoms consistently in the past three months(*P* = 0.002), ever have group sex with males (*P* < 0.001) and commercial sex (*P* < 0.001). (Table 1).

### Coerced syphilis self-testing experience

#### Numbers and location

Among those 31 men who reported experience syphilis self-testing coercion throughout their life, 11 (35.5%) men had experienced syphilis self-testing coercion one time, 10 men (32.3%) had experienced coercion twice, and 9 (29.0%) participants had experienced coercion three times in their lifetime. And only one participant had experienced syphilis self-testing coercion four times. (Table 2).

**Table 2:** Coerced syphilis self-testing characteristics among MSM in China (*N* = 31)

Item	Number	Percentage (%)
*Numbers of syphilis self-testing coercion during their lifetime*		
1	11	35.5
2	10	32.3
3	9	29.0
4	1	3.2
*Location where syphilis self-testing coercion happened*		
Hotel	14	45.2
Other people's homes	11	35.5
Your own home	7	22.6
Medical and health department	4	12.9
Community Organization	2	6.5
Workplace	1	3.2
Entertainment venues (e.g. sauna, bath, bar)	1	3.2
Other places	1	3.2
*Relationship with the person who coerced you*		
Fixed male sexual partner	25	80.6
Casual male sexual partner	6	19.4
Fixed female sexual partner	2	6.5
Friends/companions	2	6.5
Healthcare worker	2	6.5
Casual female sexual partner	1	3.2
*Types of coercion before syphilis self-testing*		
Verbal abuse	12	38.7
Threatening to end a relationship	12	38.7
Threats of violence	9	29.0
Psychological pressure	7	22.6
Physical violence	6	19.4
Excessive control of activities	6	19.4
Withholding of household resources	3	9.7
Other	1	3.2
*Types of coercion after getting self-testing results*		
Threatening to end a relationship	14	45.2
Threats of violence	11	35.5
Physical violence	9	29.0
Verbal abuse	7	22.6
Psychological pressure	7	22.6
Excessive control of activities	3	9.7
Withholding of household resources	3	9.7
Other adverse outcome	5	16.1
Other negative outcome	2	6.5

Almost half of syphilis self-testing coercion happened in hotel (45.2%,14/31), followed by other people’s home (35.5%,11/31), own home (22.6%, 7/31), medical and health department (12.9%,4/11) and community organization (6.5%, 2/11) (Table 2).

#### Relationship with the person who coerced you

The majority (80.6%, 25/31) reported that their most recent syphilis self-testing coercion experience was coerced by their fixed male sexual partners, followed by casual male sexual partners (9.4%, 6/31) and fixed female sexual partners (6.5%, 2/31) (Table 2).

#### Types of syphilis self-testing coercion

The most common types of coercion before syphilis self-testing were verbal abuse (38.7%, 12/31) and threatening to end a relationship (38.7%, 12/31). After obtaining the self-test results, men were mostly subjected to end a relationship (45.2%, 14/31) and threaten of violence (35.5%, 11/31) (Table 2).

### Factors correlated with coerced syphilis self-testing experience

After adjusted for age, legal marital status, highest educational attainment, annual income and sexual orientation, multivariable logistic regression analysis indicated the odds of experiencing syphilis coercion were 8.08 (95%CI: 2.36–27.69) among those who using condoms inconsistently compared to consistent condom users (*P* = 0.001), were 6.70(95%CI:2.05–21.95) among those who ever used substances before or during sex compared to non-users (*P* = 0.002), were 8.30(95%CI: 2.90–23.75) among those who ever had group sex with males compared to without group sex (*P* < 0.001), were 10.22(95%CI: 3.66–28.55) among those who ever had commercial sex compared to without commercial sex (*P* < 0.001) (Table 3).

**Table 3:** Factors associated with ever experiencing syphilis self-testing coercion among MSM in China (*N* = 174)

Variable	Crude *OR*	*P*	Adjusted OR*	*P*
*Age*				
16–25	*Ref*		*Ref*	
26–35	1.91 (0.83–4.40)	0.130	1.00 (0.30–3.29)	0.999
> = 36	0.39 (0.05–3.25)	0.383	0.08 (0.01–0.91)	0.041
*Legal marital status*				
Never married	*Ref*		*Ref*	
Ever married/engaged	5.38 (2.28–12.66)	< 0.001	9.52 (3.08–29.37)	< 0.001
*Highest educational attainment*				
High school or below	*Ref*		*Ref*	
Junior college	0.52 (0.14–2.00)	0.342	0.55 (0.13–2.37)	0.423
Bachelor's degree or higher	1.60 (0.59–4.35)	0.357	1.23 (0.38–3.96)	0.731
*Annual income (US$)*				
< $5000	*Ref*		*Ref*	
$5001–9000	0.93 (0.31–2.79)	0.901	0.71 (0.21–2.42)	0.582
> = $9001	1.69 (0.64–4.50)	0.291	0.76 (0.20–2.92)	0.693
*Sexual orientation*				
Gay	*Ref*		*Ref*	
Bisexual	1.25 (0.49–3.22)	0.644	0.94 (0.33–2.72)	0.912
Other	2.00 (0.36–10.98)	0.425	1.60 (0.18–14.13)	0.674
*Disclosure of sexual orientation to family or friends or health provider*				
Yes	1.69 (0.60–4.72)	0.321	1.22 (0.40–3.74)	0.734
No	*Ref*		*Ref*	
*Number of male sex partners in the past 3 months*				
< = 1	*Ref*		*Ref*	
2–5	3.12 (1.34–7.31)	0.009	2.53 (0.97–6.62)	0.058
≥ 6	1.76 (0.18–16.74)	0.625	3.99 (0.34–46.48)	0.269
*Inconsistent condom uses with men in the past 3 months*				
Yes	9.09 (3.00–27.56)	< 0.001	8.08 (2.36–27.69)	0.001
No	*Ref*		*Ref*	
*Condom use with men in the last sexual act*				
Yes	3.36 (1.51–7.46)	0.003	2.15 (0.83–5.59)	0.117
No	*Ref*		*Ref*	
*Ever used substances before or during sex*				
Yes	7.24 (2.41–21.75)	< 0.001	6.70 (2.05–21.95)	0.002
No	*Ref*		*Ref*	
*Ever had group sex with males*				
Yes	7.22 (3.11–16.76)	< 0.001	8.30 (2.90–23.75)	< 0.001
No	*Ref*		*Ref*	
*Ever had commercial sex*				
Yes	9.45 (3.98–22.43)	< 0.001	10.22 (3.66–28.55)	< 0.001
No	*Ref*		*Ref*	
*Ever had HIV testing*				
Yes	0.64 (0.07–6.39)	0.701	0.27 (0.02–3.22)	0.300
No	*Ref*		*Ref*	
*Ever had HIV self-testing*				
Yes	1.09 (0.12–9.65)	0.940	0.45 (0.04–5.31)	0.527
No	*Ref*		*Ref*	
*Ever had facility syphilis testing*				
Yes	0.62 (0.22–1.73)	0.358	0.81 (0.27–2.47)	0.712
No	*Ref*		*Ref*	
*Syphilis infection*				
Positive	6.60 (2.53–17.22)	< 0.001	5.65 (1.76–18.08)	0.004
Negative	*Ref*		*Ref*	

*Age, legal marital status, highest educational attainment, annual income and sexual orientation were adjusted for each other; all other variables were adjusted for age, legal marital status, highest educational attainment, annual income and sexual orientation.

## Discussion

Syphilis self-testing can help expand syphilis testing uptake among MSM [5, 6]. The World Health Organization (WHO) declared that HIV and syphilis tests should be voluntary, and coerced testing is never appropriate [16]. Our study suggests that coerced syphilis self-testing was prevalent among MSM in China. This study expands the existing literature by focusing on MSM with syphilis self-testing experience, monitoring for the potential harms and exploring factors associated with coerced syphilis self-testing. Findings from this study have the potential to reduce coerced syphilis self-testing while enhancing syphilis test uptake among MSM.

We found that the syphilis self-testing coercion was prevalent in our sample of MSM in China and about half of coercion happened in hotel. The rate is higher than HIVST coercion reported by the study conducted in 2016 among 1312 MSM in China (8%) [11], Malawi (2.9%) [14] and Uganda (0.0%) [17]. This may be related to the high burden of syphilis among MSM, fear of stigma and discrimination of syphilis infection, and poor communication between sexual partners [18]. We found that the prevalence of syphilis is very high (12.6%) in the sample of MSM that completed the survey. Another qualitative study regarding to pressured HIV testing among Chinese MSM revealed that the desire to develop a relationship and lack of advanced notice of the test were also important factors [12]. Additionally, China’s relatively permissive regulatory environment on self-testing [11], unequal power in intimate relationships [19] and the increasing availability of online syphilis self-testing kits [20] may contribute as well.

We found that MSM who engaged in risky sexual behaviors were more likely to experience syphilis self-testing coercion. This finding is consistent with previous study on HIVST coercion among MSM in China [11]. This might attribute to the following reasons. First, compared with the general population, MSM have a higher willingness of using self-testing as a risk reduction technique to screen sexual partners before sex in order to ensure safe sex, especially for partners with risky behaviors who have a higher burden of syphilis infection, sometimes called “point-of-sex” testing [11]. This may lead to situations where testing is coercive in certain circumstances if their partners refuse [21]. Second, previous studies showed that MSM with high-risk sexual behaviors had lower testing self-efficacy (testing self-efficacy refers to people’s level of confidence to have testing) [22], which could result in a high likelihood of being forced to take the syphilis test. Although there is enthusiasm for using mutual partner testing to promote testing uptake and reduce unprotected sex, future programs supporting point-of-sex testing should also strengthen the monitoring of the probable negative outcomes of test coercion.

We found that the types of coercion before or after syphilis self-testing were mostly verbal and psychological (e.g., verbal abuse, threating to end the relationship), with some physical violence responses (e.g., hitting, kicking). The incidence of different forms of syphilis self-testing coercion was higher than the previous study on HIV self-testing coercion among MSM in United States [15]. Although the use of syphilis self-tests may not increase physical violence harm experienced, verbal and psychological coercion may cause MSM anxiety, stress disorder, depression, and other health complications [23]. Therefore, mental health and supportive services should be strengthened for MSM with experience of coerced syphilis self-testing. A comprehensive support structure is required to connect and coordinate existing mental health services with one another and with healthcare services.

The WHO guideline on HIVST have emphasized the necessity of implementing proper measures to monitor for possible HIVST hazards. Many developed countries have already utilized existing systems or enacted regulatory related policies (such as national health policies to regulate the sale, distribution and use of diagnostics intended for self-testing) to monitor the harms of HIVST, resulting in a reduction in the risk of coercion associated with self-testing [24–26]. Our study underscores the importance for policies to be in place to monitor for potential harms of syphilis self-testing in China. In addition, multiple studies have suggested that-community engagement, post-testing counselling and effective communication between peers can reduce syphilis self-testing coercion [12, 27, 28].

There are several limitations in this study. First, we did not interview individuals who coerced other participants to take syphilis test in our study. This is critical to investigate the reasons behind coerced syphilis testing in future study. Second, all the information in this study was gathered by voluntary self-report, which may be prone to information bias. Third, the study may have a selection bias because we recruited participants online, MSM recruited online tended to be younger and more educated [29], therefore, online recruitment might underrepresent MSM with lower levels of education. Fourth, since this was a cross-sectional study, relations should be interpreted as associations that might or might not be casual. Fifth, our study recruited participant exclusively online with a relatively small sample size, this may limit the statistical inference and generalizations of the results. However, our empirical generalizability research found that the results were similar when the online survey was quantitatively generalized to a national, cross-sectional survey dataset on MSM in China [7, 8, 30]. And according to a previous study that when sample size is 10 times greater than the number of variables, the power of the result was enough [31].

Our findings have several implications for research and policy. From a research perspective, our study expands the scarce existing data by providing a comprehensive analysis of syphilis self-testing coercion among Chinese MSM. Further research studies will be important to intentionally collect extensive information on syphilis self-testing coercion to provide evidence base for the scalability of syphilis self-testing and take effective interventions to reduce coercion. From a policy perspective, this research can help in the development of related strategies for monitoring and preventing syphilis self-testing hazards. Self-testing coercion is present in both syphilis and HIV self-testing. Hence, it is vital to consider merging the efforts to reduce syphilis and HIV self-testing coercion.

## Conclusions

The experience of coerced syphilis self-testing was prevalent among MSM in China. Policies should be in place to monitor and prevent for potential harms associated with syphilis self-testing coercion. Innovative interventions to monitor and reduce coerced syphilis self-testing are necessary among MSM in China.

## Supplementary Information

**Additional file 1.** SST Questionnaire.

## Data Availability

The datasets analyzed during this study are not publicly available due to ethical and confidentiality reasons but are available from the corresponding author on reasonable request.

## Abbreviations

MSM: Men who have sex with men
LMIC: Low- and middle-income countries
HIV: Human immunodeficiency virus
HIVST: HIV self-testing
WHO: World Health Organization
