# Supplementary material for: Coerced syphilis self-testing among men who have sex with men: a nationwide cross-sectional study in China
Source: BMC Infect Dis. 2022 May 23;22:485. doi: 10.1186/s12879-022-07476-2 (PMC9125904; doi:10.1186/s12879-022-07476-2)
Supplement: Supplementary file 1 — Additional file 1. SST Questionnaire. [file 12879_2022_7476_MOESM1_ESM.docx]

**Eligibility Survey (Q1-3)**

1. Were you born biologically male or female?

- Male
- Female (Not eligible to take this survey – Skip to End of Survey)

2. What is your date of birth?

- dd.mm.yyy (*Calendar input*) (Not eligible to take this survey if year is greater than Launch day + 2002 or < 16 y/o – Skip to End of Survey)

3. In your lifetime have you ever had anal / oral sex with another man?

- Yes
- No (Not eligible to take this survey – Skip to End of Survey)

**A. Sociodemographics**

*These questions will ask you to provide some information about yourself.*

A1. What province or province-level city do you currently live in?

- Beijing
- Tianjin
- Hebei
- Shanxi
- Inner Mongolia
- Liaoning
- Jilin
- Heilong Jiang
- Shanghai
- Jiangsu
- Zhejiang
- Anhui
- Fujian
- Jiangxi
- Shandong
- Henan
- Hubei
- Hunan
- Guangdong
- Guangxi
- Hainan
- Chongqing
- Sichuan
- Guizhou
- Yun An
- Xizang (Tibet)
- Shaanxi
- Gansu
- Qinghai
- Ningxia
- Xinjiang
- Hong Kong
- Aomen

A2. What city do you currently live in? _____________ (*Text input)* (Do not display if answered Beijing,Shanghai,Chongqing,Tianjin,Hongkong,Macau to A1）

A3. What is your current legal marital status (referring to women)?

- Never married
- Engaged or Married
- Separated or Divorced
- Widowed

A4. Are you currently enrolled as either a full-time or part-time student?

- Yes
- No

A5. What is the highest level of education that you have **completed**?

- High school or below (including Zhongzhuan)
- Some college (Dazhuan)
- College/Bachelors
- Masters
- PhD

A6. What is your total individual **monthly** income from all sources?

- Less than 1500 RMB
- Between 1500 and 3000 RMB
- Between 3001 and 5000 RMB
- Between 5001 and 8000 RMB
- Greater than 8000 RMB

A7. What do you primarily identify as?

- Gay
- Bisexual
- Straight/Heterosexual
- Transgender
- Unsure/Other

A8. Have you ever spoken with a physician or other health professional (e.g. HIV testing counselor, pharmacist) about your sexual orientation?

- Yes
- No

A9. Have you ever spoken with your family or a non-gay friend about your sexual orientation?

- Yes
- No

**B. Sexual Behavior**

*The next set of questions will ask you about your sexual behaviors*

Note: In this section, when we ask about sexual behavior with male partner, this refers to anal sex only.

B1. In the last three months, approximately how many male sex partners have you had?

_________male sex partners *(Number input)* (If answer <1, skip to B7)

B2. The last time you had anal sex with a male partner, did you use a condom?

- No
- Yes

B3. In general, when you have anal sex with a male partner, how frequently do you use a condom?

- Never used
- Sometimes used
- Mostly used
- Always used

B4. In the last three months, when you had anal sex with your male partner, what role did you assume?

- Always insertive (always 1)
- Mostly insertive (mostly 1)
- Mostly receptive (mostly 0)
- Always receptive (always 0)
- Both insertive and receptive in similar amounts (Both 1 and 0 in similar amounts)

B5. In the last three months, when you had sex with your male partner, how frequently did you or your partner use condoms?

- Never used
- Sometimes used
- Mostly used
- Always used

B6. In the last three months, how often did you have sex while you and/or your partner were drunk?

- Never
- Rarely
- Occasionally/Sometimes
- Very often
- Always

B7. In the last three months, approximately how many female sex partners have you had?

_________female sex partners *(Number input)* (If answer <1 then skip to B9)

B8. In the last three months, when you had sex with your female partner, how frequently did you or your partner use condoms?

- Never used
- Sometimes used
- Mostly used
- Always used

B9. Have you ever used the following substances before or during sex? (select all that apply)

- None (Cannot be selected at the same time as other options)
- Rush poppers
- Capsule ‘0’
- G point liquid (G点液)
- Viagra or viagra products
- Heroin/Morphine/Opium
- Marijuana
- Methamphetamine/ Crystal meth/ Ecstasy / Smurfs / White angel / Butterfly / Happy Water / "jumping sugar" / bath salt
- Others (______)

B10. Have you ever participated in group sex with other men?

(Group sex is defined as have sex with two or more men simultaneously.)

- Yes
- No

B11. Have you ever paid (with money or gifts) a man/woman to have sex?

- A man
- A women
- Both
- Neither

B12. Have you ever received money or gifts to have sex with a man/woman?

- A man
- A women
- Both
- Neither

**C. HIV Testing**

*The next set of questions will ask about your HIV testing and results. Self-testing refers to you administering the test yourself and interpreting results yourself.*

C1. Have you ever been tested for HIV?

- Yes
- No (Skip to Section D)

C2. Have you ever self-tested for HIV?

- Yes
- No (Skip to C4

C3. Where did you obtain your HIV self-test kit? (select all that apply)

- online pharmacy store (Taobao, JD, Amazon)
- hospital
- pharmacy
- Community based organization (CBO) (include ordering HIVST kit from CBO official website or service platform)
- Friend
- Family member
- Other _________________

C4. What was the result of your most recent HIV test?

- HIV negative/uninfected (skip to D1)
- Screen HIV positive (never done confirmation test) (skip to D1)
- Confirmed HIV positive/infected
- I never got my test results (skip to D1)

C5. Following your positive/ indeterminate result, did you see you doctor?

- Yes
- No

C6. What was the time between your positive HIV test and seeking for ART care?

- 0-2 weeks
- 2-4 weeks
- 1-3 months
- >3 months
- I am not currently in ART

**D. Syphilis Testing**

*The next set of questions will ask about your syphilis testing and results. Self-testing refers to you administering the test yourself and interpreting results yourself. A “regular partner” is someone who you have sex with regularly, have an emotional commitment to, and/or have married or engaged to be married.*

D1. Have you ever been tested for syphilis (include tested in the facilities such as hospital, clinic and CBO, and self-test)?

- Yes
- No (Skip to Section 4)

D2. Have you ever had syphilis test through the following channels? (Check all that apply)

- Public hospital/STD specialist hospital
- Private hospital
- CDC (Voluntary counselling HIV test site)
- Community health centre
- Community based organization
- Blood centre
- Used a self-test kit
- Other ( )

D3. Do you know your result of the last test?

- Positive
- Negative
- Indeterminate
- I don’t know

D4. Have you ever used a self-testing kit for syphilis?

- Yes (Skip to D6)
- No (Do not display D6-D37, Skip to D5)

D5. What were your reasons for not using syphilis self-testing (select all that apply)?

- Have already tested at a clinic
- Haven’t heard of syphilis self-testing
- Worry about the accuracy of the test result
- Afraid of cannot correctly determine the result by themselves
- Afraid of taking blood
- Not familiar with operating instructions
- The cost is too high
- Worry about the test kit may expose my privacy
- Don’t know how to obtain it
- Other _____________

D6. Have you ever performed syphilis and HIV self-testing together?

- Yes
- No (skip to D8)

D7. What kind of HIV and syphilis self-test kit did you use for your last self-test?

- HIV and Syphilis dual test kits or other combo test kits (eg HIV, syphilis, HCV and HBV)
- Separate HIV and syphilis test kit

D8. How hard was it for you to follow the self-test instruction?

- Very difficult
- Somewhat difficult
- Slightly difficult
- Slightly easy
- Somewhat easy
- Very easy

D9. Which step do you find difficult for you to take as you test (select all that apply)?

- None (Cannot be selected at the same time as other options)
- Understanding the instructions for performing the test
- Removing lancet cap
- Pricking finger
- Squeezing the finger firmly to extract blood
- Using collection tube to collect blood
- Timing the test
- Interpreting the results

D10. Was your self-test the first time you ever tested for syphilis?

- Yes
- No

D11. What happened to your syphilis testing frequency after you first used a self-test?

- Increased
- Decreased
- No change

D12. Where did you obtain your syphilis self-test kit? (Select all that apply)

- Online pharmacy store (Taobao, JD, Amazon)
- Hospital
- Pharmacy
- Community based organization (CBO) (include order self-test kit from CBO official website or service platform)
- Friend
- Family
- Other___________________

D13. What was your reason for performing the syphilis self-test? (Select all that apply)

- I wanted to know my syphilis status
- I had symptoms I was worried was due to syphilis
- Recently had high risk sexual behavior
- I was recommended to test by healthcare staff
- My regular partner asked me to test
- A casual partner asked me to test
- I wanted to be the first person to read my test
- Other ________________

D14. If you have extra syphilis self-testing kits on hand, who do you want to use with? (Select all that apply)

- Regular partner
- Casual partner
- Fuckbuddy
- Gay friend/s (without sexual relationship)
- None

D15. What was the result of your most recent syphilis self-test?

- Positive
- Negative (skip to D24)
- Indeterminate

D16. Following your positive/ indeterminate syphilis self-test, did you see a doctor?

- Yes
- No(Skip to D21)

D17. Where did you seek care?

- Local general hospital
- Specialist hospital for STD
- Pharmacy (brought drugs by yourself)
- CDC
- Online consultation
- Other ______________________

D18. What was the time between your syphilis self-test result and seeking care?

- 0-2 weeks
- 2-4 weeks
- 1-3 months
- >3 months

D19. Did the care provider repeat a test for syphilis?

- Yes
- No

D20. Did you complete treatment for syphilis?

- Yes (Skip to D24)
- No (Skip to D24)

D21. What were your reasons for not attending a health care provider after your syphilis self-test?

- I knew it was past infection and I have already been treated.
- Don’t believe the result
- No time to attend clinic
- Unable to afford money for transport to clinic
- Don’t want to be seen at STI clinic (e.g. because of shame)
- others (_____________)

D22. Following your positive/ indeterminate syphilis self-test, did you obtain treatment for syphilis without attending a hospital/clinic?

- Yes
- No (Skip to D24)

D23.Where did you obtain treatment for syphilis?

- Private Doctor
- Traditional Medicine Practitioner
- Brought drugs form drug store by yourself
- Internet
- Other _____________

D24.Would you use syphilis self-test again in the future?

- Yes
- No

***Note: The current syphilis treatment guideline is unable to accept self-test results as sufficient cause to prescribe treatment. Hence the need for retesting at the clinic before you can obtain syphilis treatment.***

D25. If you did not need to also be tested at clinic to obtain treatment, would you be more likely to self-test?

- Yes, much more likely to self-test
- Yes, a bit more likely to self-test
- No more likely to self-test

D26. Did your regular partner know you had undertaken a syphilis self-test?

- Yes
- No
- I did not have a regular partner when I self-tested

D27. Did your regular partner also undertake a syphilis self-test?

- Yes
- No
- Don’t Know
- I did not have a regular partner when I self-tested

D28. We would like to confirm that you undertook a syphilis self-test. Please send us one of the following four items to confirm your self-testing. If you send us proof of confirmation, we will provide 20 RMB **credit** to your mobile phone. Confirmation proof includes one or multiple of the following:

- Photograph of the receipt you used to purchase your self-test kit, with your name covered or obscured for personal privacy.
- Screenshot of the WeChat transaction showing that you purchased a self-test kit, with your name covered or obscured for personal privacy.
- Photograph of your used syphilis self-test kit.

Please leave your WeChat information here: _________(We will grant the money for you after verifying your proof materials.)

***The next set of questions is about pressured syphilis testing. Pressured testing is when someone pressures you against your will to do a syphilis test by using verbal, physical or psychological threat. Pressure may be through physical means or could involve threats to take away something if you don’t do the test (e.g. losing your job, breaking up a relationship, not having sex)***

(The following questions are depended on the question of D1 and D4. Participant who answered “Yes” to either D1 or D4, is needed to complete the following questions.)

D29.In your life, how many times have you been pressured/forced into taking a syphilis test? ______ (If answer <1, skip to end of this section)

D30.How many times did you use the following type of syphilis test as a result of the pressure?

|  | **Times** |
| --- | --- |
| Rapid syphilis test provided by a healthcare professional | **€** |
| Rapid syphilis test provided by a peer or friend or family member who is not a healthcare professional | **€** |
| Rapid syphilis test that you bought yourself | **€** |
| Non-rapid facility based test | **€** |
| Other (type of test) | **€** |

D31.In your most recent experience of pressured syphilis test, who pressured you to test for syphilis? (select all that apply)

- Regular Male Sexual Partner
- Casual Male Sexual Partner
- Regular Female Sexual Partner
- Casual Female Sexual Partner
- Friends/Peers
- Healthcare Worker
- Manager/Boss/Employer
- College/University/Educational institution
- Civil Servant/Government officials
- Parent(s)
- Other family members
- Other (person)

D32.Was the person who pressured you present when you performed the test?

- Yes
- No

D33.Before you took your syphilis test, from the person who pressured you to take the test, did you experience the following items? (select all that apply)

- Actual physical violence (e.g. pushing, slapping, punching, kicks)
- Threats of physical violence
- Verbal abuse (e.g. being shouted at)
- Psychological abuse (e.g. being ignored)
- Being controlled (e.g. not allowed to leave the house)
- Denying access to household resources (e.g. food, money)
- Threaten for relationship to break up
- Other_______________________

D34. Where were you pressured to test for syphilis? (tick all that apply)

- Your home
- Someone else’s home
- Workplace or Danwei
- Hotel
- College/University/Educational institution
- Detainment center
- Health-care Facility
- Community Based Organization
- Entertainment venue (e.g. sauna bath, bar)
- Other (place)

D35.The last time you were pressured to test for syphilis, what test was used?

- Rapid syphilis test provided by a healthcare professional
- Rapid syphilis test provided by a peer or friend or family member who is not a healthcare professional
- Rapid syphilis test that you bought yourself
- Non-rapid facility based test (i.e. blood taken from your vein)
- Other (type of test)

D36.What was your test result?

- Positive
- Negative
- Indeterminate
- I don’t know

D37.After you received your syphilis test results, from the person who pressured you to take the test, did you experience the following items?

- Being physically hurt
- Threats of being physically hurt
- Verbal insult
- Psychological insult
- Being controlled
- Denying access to household resources
- Threaten for relationship to break up
- Other negative outcome?
- Other positive outcome?

**E. Risk Aversion Scale**

E1.How do you see yourself: are you generally a person who is fully prepared to take risks or do you try to avoid taking risks? Place tick a box on the scale, where the value 0 means “not at all willing to take risks” and the value 10 means “very willing to take risks”.

| How do you see yourself: | 0 | 1 | 2 | 3 | 4 | 5 | 6 | 7 | 8 | 9 | 10 |
| --- | --- | --- | --- | --- | --- | --- | --- | --- | --- | --- | --- |
| 1. in **general** |  |  |  |  |  |  |  |  |  |  |  |
| 2. in **finances** |  |  |  |  |  |  |  |  |  |  |  |
| 3. in **sports and leisure** |  |  |  |  |  |  |  |  |  |  |  |
| 4. in **career** |  |  |  |  |  |  |  |  |  |  |  |
| 5. in **health** |  |  |  |  |  |  |  |  |  |  |  |
| 6. in **car driving** |  |  |  |  |  |  |  |  |  |  |  |

E2.How likely would you test for syphilis in the coming 3 months?

- Very unlikely
- Unlikely
- Neither likely nor unlikely
- Likely
- Very likely

E3.If there is an opportunity to participate in a lottery (i.e. 1 in 10 chance to win a money prize if you test syphilis-negative), how likely would you test for syphilis in the coming 3 months?

- Very unlikely
- Unlikely
- Neither likely nor unlikely
- Likely
- Very likely

E4.What is the minimum amount of money in the lottery that would attract you to participate to test for syphilis in the coming 3 months? _______ RMB
